# High Density Lipoprotein Assessment Revisited: A Review of Foundational Understanding and Recent Developments in Function, Particle Size, and Cardiometabolic Impact

**DOI:** 10.1007/s11883-026-01413-3

**Published:** 2026-04-25

**Authors:** Anita Sherly A, Rukmini Mysore Srikantiah, Anand Rohatgi, Anupama Hegde, Sindhu H, Arun S

**Affiliations:** 1 Department of Biochemistry, Kasturba Medical College Mangalore, Manipal Academy of Higher Education, Manipal, India; 2 Department of Medicine, Division of Cardiology, UT Southwestern Medical Centre, Dallas, TX USA; 3 Department of Medicine, Kasturba Medical College Mangalore, Manipal Academy of Higher Education, Manipal, India

**Keywords:** High density lipoprotein, Cardiovascular disease, Nuclear magnetic resonance, Cholesterol efflux capacity, Apolipoproteins

## Abstract

**Purpose of Review:**

High density lipoprotein (HDL) has historically been considered cardioprotective, with HDL cholesterol (HDL-C) levels widely used as a biomarker of cardiovascular disease risk. However, accumulating evidence suggests that HDL-C concentration alone does not adequately capture the complex biological functions of HDL particles. This review revisits the evolving understanding of HDL biology and examines the emerging importance of HDL functionality, particle size and particle number in cardiometabolic risk assessment. It further highlights recent methodological advances that enable more detailed characterization of HDL heterogeneity.

**Recent Findings:**

Contemporary research demonstrates that HDL represents a structurally and functionally heterogenous group of particles with diverse biological activities. Cardioprotective effects attributed to HDL are increasingly linked to functional properties rather than to HDL-C levels alone. Growing evidence suggest that HDL particle concentration and subclass distribution may correlate more strongly with cardiovascular outcomes than conventional HDL-C measurements. Technological advances, such as nuclear magnetic resonance(NMR) spectroscopy and polyacrylamide gel electrophoresis based systems like the Lipoprint platform, now allow more precise quantification and classification of HDL subfractions.

**Summary:**

Current evidence challenges the traditional reliance on HDL-C as a surrogate marker of cardiovascular protection. Instead, HDL should be viewed as a dynamic and heterogenous lipoprotein system in which particle characteristics and functional capacity are critical determinants of cardiometabolic risk. Incorporating measurement of HDL particle distribution and functionality into research and clinical evaluation may improve cardiovascular risk stratification and deepen understanding of HDL biology. Continued advances in analytical technologies and functional assays are likely to reshape HDL assessment and support more precise, mechanism based approaches to cardiovascular disease prevention and management.

## Introduction

High-density lipoprotein- (HDL) has traditionally been considered a protective factor against cardiovascular disease (CVD) due to its role in reverse cholesterol transport (RCT), wherein excess cholesterol is removed from peripheral tissues and transported to the liver for excretion [1]. Epidemiological studies have consistently shown that HDL cholesterol (HDL-C) levels have an inverse relationship with CVD risk, suggesting that higher HDL-C levels confer cardio protection [2, 3]. However, recent research challenges this simplistic view, disproving the notion of a single silver bullet by emphasizing that HDL functionality, rather than just HDL-C concentration, determines its atheroprotective potential.

Despite the initial promise of HDL-targeted therapies, clinical trials using cholesteryl ester transfer protein (CETP) inhibitors and niacin to raise HDL-C levels failed to show a corresponding reduction in cardiovascular events [4, 5]. These findings suggest that HDL-C is an inadequate surrogate for HDL function. Consequently, the focus has shifted towards understanding qualitative aspects of HDL, such as its cholesterol efflux capacity (CEC), antioxidative and anti-inflammatory properties, endothelial function, and proteomic composition [6]. Among these, CEC, the ability of HDL to facilitate cholesterol removal from macrophages via ATP-binding cassette transporter A1 (ABCA1), has emerged as a stronger predictor of cardiovascular risk than HDL-C [7].

Various subclasses of HDL differ in size, density, and composition, making it a heterogeneous lipoprotein. The presence of larger HDL particles, particularly HDL2, is often associated with enhanced cholesterol efflux and anti-inflammatory properties, while smaller HDL particles exhibit reduced functionality [8]. The use of techniques like nuclear magnetic resonance (NMR) spectroscopy and Lipoprint analysis [9] has enabled a more refined characterization of HDL subclasses due to recent advancements in lipidomic and proteomics. The comprehensive evaluation of HDL biology provided by these methods reveals that alterations in HDL particle distribution and functionality may be more accurate than HDL-C levels alone in assessing cardiovascular risk [10].

The aim of this narrative review is to explore the changing paradigm of HDL function, the clinical significance of HDL subclass characterization, and the implications for cardiovascular risk assessment. By integrating emerging insights from lipidomic, proteomics, and advanced lipid profiling techniques, we underscore the need to transition from an HDL-C-centric approach to a more comprehensive evaluation of HDL metabolism in cardiovascular disease prevention and management by addressing metrics of HDL function.

## HDL – Knight in Shining Armor

**Fig. 1:**
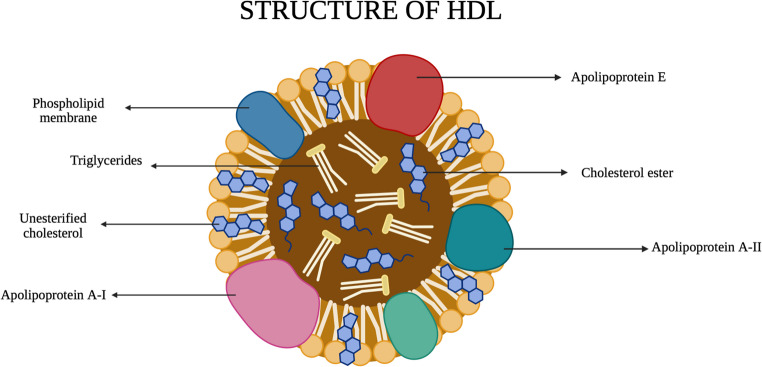
HDL Description: HDL is a complex particle composed of lipids and proteins. It consists of a lipid core surrounded by a phospholipid monolayer and apolipoproteins. The apolipoproteins present include apolipoprotein A1, A2 and Apolipoprotein E on the surface

Lipoproteins are vital biological entities that transport lipids across cellular and tissue barriers while also mediating intracellular signalling pathways. Their structure comprises a hydrophobic core that is made up of esterified cholesterol and triacylglycerol, which is surrounded by a phospholipid monolayer that contains unesterified cholesterol, along with apolipoproteins embedded on the surface [10, 11]. These lipoproteins are broadly classified into five major subtypes: Very Low-Density Lipoprotein (VLDL), Intermediate-Density Lipoprotein (IDL), Chylomicrons (CL), Low-Density Lipoprotein (LDL), and High-Density Lipoprotein (HDL), each differing in size, lipid (triglyceride and cholesterol) composition, density, and protein content [12, 13]. Among these, HDL is the smallest (7–12 nm) and most protein-rich (45–55%) lipoprotein, with the highest protein-to-lipid ratio (Fig. 1). Proteins are the primary component of HDL, unlike other lipoproteins [14]. In the 1950 s, the association between HDL-C levels and risk of coronary heart disease (CHD) was first identified, and later studies from the 1970 s onward reinforced this relationship. Consequently, HDL has earned the label ‘good cholesterol’, in contrast to LDL, which is often deemed ‘bad cholesterol’ [15].

As a plasma cholesterol carrier, HDL has traditionally been studied as a protective anti-atherogenic and anti-inflammatory property. Systemic or vascular inflammation can cause structural and functional changes in HDL, causing it to become dysfunctional and promote atherosclerosis rather than preventing it [15, 16]. Recent research has moved beyond the quantity of HDL to focus on its quality, as its functional properties depend on its protein and lipid composition. The most abundant protein in HDL is apolipoprotein A-I (apoA-I), accounting for approximately 70% of its total protein content. ApoA-I plays a crucial role in activating lecithin-cholesterol acyltransferase (LCAT), which facilitates anti-atherogenic processes, and in interacting with cellular receptors [17]. The second most prevalent HDL-associated apolipoprotein is apolipoprotein A-II (apoA-II), which makes up around 15–20% of HDL’s protein content [18, 19], while the remaining 10–15% consists of various minor apolipoproteins and enzymes, including apoA-IV, apoCs (enzyme regulators), apoD, ApoE, apoF, apoH, apoJ, apoL-I, and apoM [20].

Paraoxonase 1 (PON1), an enzyme closely linked to HDL, is well known for its strong anti-inflammatory and antioxidative properties [21]. In addition, HDL is connected to other essential enzymes like platelet-activating factor acetylhydrolase (PAF-AH) and LCAT. HDL function is regulated by Cholesteryl ester transfer protein (CETP) and phospholipid transfer protein (PLTP), both of which are essential for lipid metabolism and transfer between lipoproteins [22, 23]. The role of apolipoproteins, which are key regulators of cholesterol metabolism and lipid transport, is crucial in both lipid metabolism and atherogenesis. Clinically significant apolipoproteins include apolipoproteins B-100, B-48, A-I, C-II, C-III, and E [18, 24]. Apolipoprotein B-48, which is a shorter version of apoB-100, is the major structural component of chylomicrons, and apoB-100 plays a crucial role in IDL, LDL, VLDL, and lipoprotein(a) [25].

**Fig. 2:**
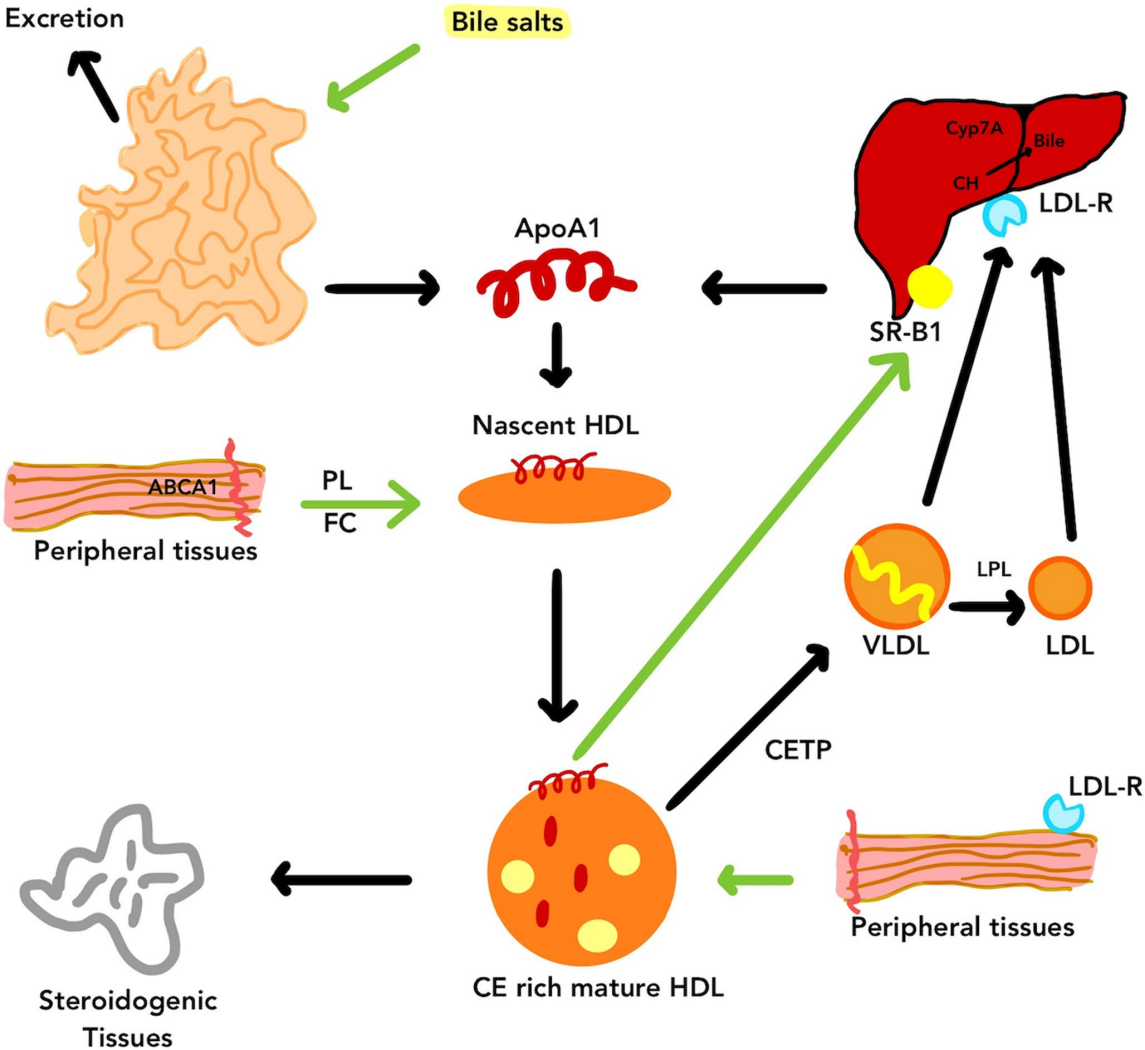
HDL Metabolism Description: Apolipoprotein A-I, produced primarily by the liver and intestines, initiates the formation of nascent HDL particles. These discoidal HDL structures acquire cholesterol and phospholipids from peripheral tissues through an ABCA1-dependent mechanism, gradually transforming into cholesterol ester-rich mature HDL. These mature HDL particles participate in reverse cholesterol transport (RCT) by delivering cholesterol to the liver. The scavenger receptor class B type 1 (SR-B1) on hepatocytes facilitates the uptake of cholesterol esters from HDL. Once inside the liver, cholesterol is metabolized into bile acids by the enzyme cholesterol 7α-hydroxylase and subsequently excreted via the intestines

**Fig. 3:**
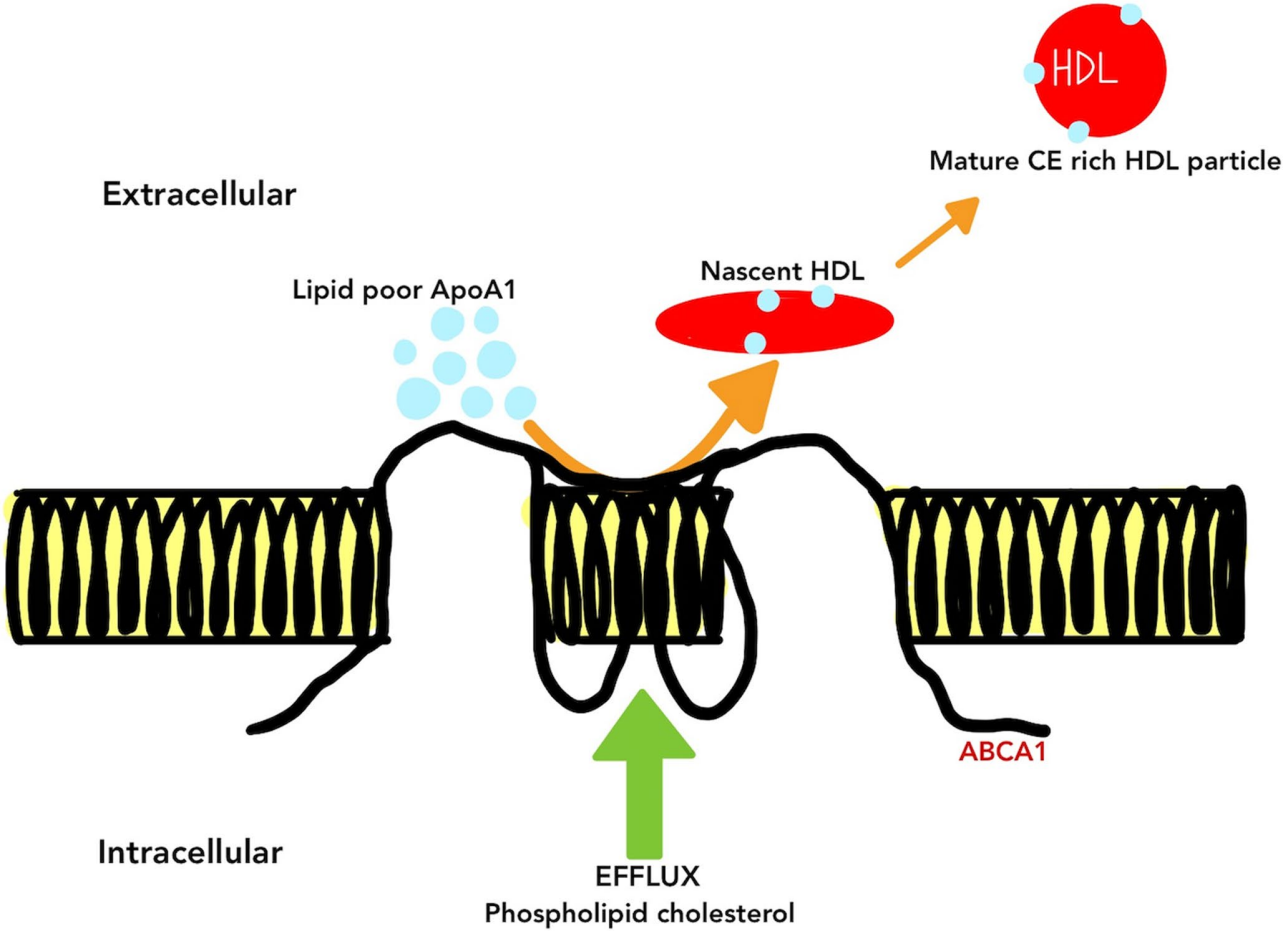
ABCA1-Mediated HDL Formation and Cholesterol Efflux Description: In the bloodstream, lipid-poor HDL particles act as acceptors of cholesterol and phospholipids from cells. This transfer is facilitated by ATP-binding cassette transporter A1 (ABCA1), a membrane-associated protein critical for initiating HDL formation. Through this process, nascent HDL particles mature into cholesterol-rich forms capable of transporting lipids to the liver. When ABCA1 function is impaired or defective, cellular cholesterol accumulates due to the inability to efficiently export lipids, leading to cholesterol deposition in tissues

ApoA-I is essential for the maturation and reverse cholesterol transport (RCT) of HDL, which are fundamental processes in cholesterol homeostasis [26]. Among the first apolipoproteins to be identified, it is still crucial for the biogenesis and function of HDL. The liver is responsible for 80% of apoA-I production, while the intestine accounts for the remaining 20% [27, 28]. The development of evidence suggests that HDL functionality is more important for cardiovascular health than HDL-C levels alone(Figs. 2 and 3). This has resulted in the development of more sophisticated methods for evaluating HDL subclasses and their functional properties. A more comprehensive understanding of HDL particle composition is expected to be facilitated by advances in proteomics and lipidomics, leading to precision-targeted therapeutic strategies.

HDL extends its protective role beyond reverse cholesterol transport (RCT) to exert vasoprotective and immunomodulatory effects that contribute to cardiovascular health. One of its critical functions is enhancing endothelial function by stimulating endothelial nitric oxide synthase (eNOS), leading to increased nitric oxide (NO) bioavailability [29]. This not only promotes vasodilation, but also exerts anti-inflammatory, anti-apoptotic, and anti-thrombotic effects, preserving vascular integrity [30]. HDL regulates signalling pathways that reduce inflammation by modulating immune cell function through influencing the cholesterol content in lipid rafts. The inflammatory cascade leading to atherosclerosis is limited by its ability to downregulate endothelial adhesion molecules and limit leukocyte adhesion and transmigration. In addition, HDL acts as a strong antioxidant by preventing the oxidation of low-density lipoprotein (LDL), which is a major factor in endothelial dysfunction and plaque formation. These multifaceted properties highlight HDL’s role in maintaining endothelial health, mitigating oxidative stress, and modulating immune response [31, 32].

### HDL Cholesterol Levels – an Important Measure of Heart Health

The development of atherosclerotic cardiovascular disease (ASCVD) has a negative correlation between HDL levels, as demonstrated by epidemiological studies. The findings of these investigations suggest that HDL could have protective effects against ASCVD, and it is identified as a significant cardiovascular risk modifier [33, 34]. However, doubts have emerged regarding the role of HDL-C as a direct mediator of ASCVD risk, particularly due to the failure of HDL-C-raising drugs to effectively reduce cardiovascular events in clinical trials [35].

The underlying precursor to heart attacks and strokes is atherosclerosis. The cholesterol hypothesis of atherosclerosis development was based on initial findings that demonstrated the accumulation of cholesterol in arterial plaques. Epidemiological investigations have established a direct correlation between elevated LDL cholesterol levels, apolipoprotein B-100 (the principal constituent of LDL), and an increased risk of ASCVD [36].

The Framingham Heart Study has been instrumental in identifying cardiovascular risk factors, which is important. The major causes of heart disease include smoking, high cholesterol, obesity, high blood pressure, physical inactivity, and diabetes. Additional studies have highlighted the significance of sex, age, socioeconomic status, and family history in cardiovascular risk assessment [37]. However, Mendelian randomized studies and numerous clinical trials have challenged the direct causal relationship between plasma HDL-C levels and CVD, as many trials have failed to demonstrate a therapeutic benefit from HDL-C-raising interventions. However, recent studies that involved larger cohorts and a higher CVD incidence have reinforced the connection between HDL-C levels and the risk of cardiovascular disease [38].

A study showed that normal HDL-C levels were present in approximately 43% of men and 44% of women who experience a myocardial infarction. Furthermore, there has been no evidence of a direct causal link between HDL cholesterol levels and the risk of coronary artery disease (CAD) in genetic investigations. Women typically have higher HDL-C levels due to hormonal influences, particularly estrogen which increases HDL synthesis(Table 1). Estrogen, acting primary through estrogen receptor α (ERα), transcriptionally regulates several lipid-related genes including ApoA1, hepatic lipase, LDL receptor, SCARB1, CETP and ApoE, collectively modulating HDL biogenesis, remodelling, and reverse cholesterol transport [39].

**Table 1:** **Genes involved in Estrogen-Regulated Lipoprotein Metabolism:** Summary of key genes involved in lipoprotein metabolism, their protein products, and the regulatory effects of estrogen, highlighting their impact on HDL biogenesis, catabolism and lipid transport pathways

Gene	Protein	Effect of estrogen	Functional consequence
APOA1	ApoA-1	↑ Upregulated	↑ HDL biogenesis
LIPC	Hepatic Lipase	↓ Suppressed	↓ HDL catabolism → ↑ HDL-C
LDLR	LDL receptor	↑ Upregulated	↑ LDL clearance
SCARB1	SR-BI	Modulated	Hepatic HDL cholesterol uptake
CETP	CETP	Modulated	Cholesteryl ester transfer
APOE	ApoE	↑ Upregulated	Lipoprotein remnant clearance

The antioxidant and anti-inflammatory properties help maintain HDL functionality by preventing oxidative modification of HDL (oxHDL), which has been mechanistically linked to impaired cholesterol efflux and clinically associated with increased ASCVD risk [40, 41].Collectively, these mechanisms elevate HDL levels, which in turn contribute to cardiovascular protection. However, higher HDL-C levels do not confer absolute immunity against CAD, highlighting the complexity of HDL functionality and cardiovascular risk [42].

This raises a critical question: If HDL-C levels are within the normal range, why do they sometimes fail to provide cardiovascular protection? The formation of HDL is a highly intricate process involving multiple enzymes, apolipoproteins, lipid transporters, cell surface receptors, and lipid transfer proteins. Collectively, these factors control HDL metabolism and have an impact on plasma HDL levels. Due to this complexity, HDL particles exhibit significant heterogeneity in terms of size, composition, and density. Thus, plasma HDL-C concentration alone may not accurately reflect its functional properties, such as its ability to mediate reverse cholesterol transport (RCT) or exert antioxidative and anti-inflammatory effects [42, 43].

## Unlocking HDL’s Hidden Depths: Beyond Face Value

We must prioritize transitioning our attention away from solely evaluating HDL levels towards conducting a thorough examination of the functional characteristics of HDL, involving CEC (cholesterol efflux capacity) and particle size. An essential role of HDL is its capacity to facilitate RCT, which includes removing surplus cholesterol from peripheral cells and transporting it to the liver for elimination. This mechanism is widely recognized as the primary anti-atherogenic function of HDL [42].

Three distinct pathways for cholesterol efflux have been identified: (1) efflux to mature HDL particles facilitated by ATP-binding cassette transporter G1 (ABCG1); (2) Efflux to lipid-free apolipoproteins, predominantly apoA-I, facilitated by ATP-binding cassette transporter A1 (ABCA1); and (3) efflux to mature HDL particles facilitated by passive diffusion as well as scavenger receptor class B type I (SR-BI) [44, 45]. Only a small fraction of circulating apoA1exists in a lipid-poor or pre-β HDL form (typically < 5–10% of total apoA-1), which is the principal acceptor for ABCA-1 mediated cholesterol efflux. The unidirectional flux mediated by ABCA1 as well as ABCG1 leads to the net elimination of cholesterol from cells. The ABCA1 pathway has been identified as crucial for maintaining cholesterol levels in normal tissue among these pathways [45]. The transmembrane protein ABCA1, comprising 2261 amino acids, plays a crucial role in the rate-limiting step of HDL biogenesis by transporting increased free cholesterol as well as phospholipids from the cell to an acceptor apolipoprotein. ABCA1-mediated lipid efflux mechanisms and the anti-inflammatory response are both triggered by the interaction between apoA-I and ABCA1 [46].

Mechanistic studies using cell-based models have clearly demonstrated that HDL mediated cholesterol efflux, particularly via the ABCA-1 pathway, plays a causal role in reverse cholesterol transport and macrophage cholesterol homeostasis. In contrast, the relationship between cholesterol efflux capacity and cardiovascular outcomes in human population is supported primarily by observational and cohort studies, in which higher CEC is associated with lower incident cardiovascular events [47, 48].While these findings strongly support the biological relevance of CEC, they do not establish direct causality between enhanced efflux capacity and reduced cardiovascular risk in humans.

The process of RCT begins when the liver or intestine releases lipid-poor apoA-I into the circulation [22, 49]. After that, it travels to peripheral cells to eliminate additional cholesterol and generate nascent HDL. The interaction between apoA-I and ABCA1 is a crucial phase in this process. Studies have shown that ABCA1 mostly enhances small HDL, known as apoA-I, to produce nascent HDL, while ABCG1 promotes cholesterol outflow to mature HDL instead of lipid-poor apoA-I [27, 47].The concept of cholesterol efflux encompasses both passive cholesterol diffusion from cells and active cellular cholesterol transfer facilitated by ABCA1, ABCG1, and SR-BI. Mature HDL is created when cholesterol is esterified by LCAT after being ingested. HDL-associated cholesteryl esters are partially transferred by CETP to triglyceride-rich lipoproteins, which are then eliminated by the liver via routes involving the LDL receptor or SR-BI [15, 48]. Consequently, cholesterol is transferred from peripheral cells to the liver through direct uptake via SR-BI or indirect interactions between HDL and LDL/VLDL. Cholesteryl esters are hydrolysed in the liver, and the free cholesterol is then either transformed into bile acids, expelled into bile via ABCG5 and ABCG8 for excretion in the faeces, or used to make VLDRs [49–51]. While LCAT facilitates HDL maturation via cholesterol esterification, kinetic studies indicate that most plasma free cholesterol is rapidly cleared within 9 min, suggesting that LCAT plays a limited role in cholesterol clearance and that earlier steps such as cellular cholesterol efflux may be more critical determinants of RCT efficiency [52].

The efflux or reverse transport mediated by ABCA1 transporter is predominantly apoA-1 dependent and represents a key mechanistic pathway in nascent HDL formation and early reverse cholesterol transport. Experimental studies clearly demonstrate that lipid-poor apoA-1 is the principal acceptor of cellular cholesterol via ABCA1, establishing a direct causal link between apoA-1 functionality and macrophage cholesterol homeostasis. In contrast, CEC mediated pathway including ABCG-1 and SR-BI primarily involves mature HDL particles and occurs largely independently of apoA-1 availability.

In recent years, a growing number of people have expressed interest in learning more about HDL-mediated cholesterol efflux. To evaluate the ability of HDL to promote cholesterol efflux or the first stage of RCT, numerous cell-based assays have been developed. Among the most well-known assays is the use of the J774 mouse macrophage line. These cells are driven to express more ABCA1, a crucial transporter involved in cholesterol efflux, after being loaded with radioactively or fluorescently labelled cholesterol. These assays measure the ratio of labelled cholesterol in the supernatant to within the cells by introducing either isolated HDL or serum depleted of apoB from patients into the cell medium [51–53].

It makes sense that there are several subfractions of ways to further divide HDL into distinct structural or functional categories given its complex composition. The primary goal of this endeavour was to identify HDL subfractions that might have diagnostic significance in predicting CVD risk. These findings have significant implications for the development of drugs that modulate HDL to prevent CVD or other related conditions. At first, HDL was classified by density, distinguishing between subfractions of HDL2 that were lighter and larger and subfractions of HDL3 that were denser and smaller. While the general process of producing HDL is similar, the specific composition and distribution of HDL subfractions c vary among individuals due to genetic, lifestyle, and health-related factors [54, 55]. Classifying HDL subfractions a to the presence or absence of apoA-II, the second most prevalent protein in HDL, was another early classification approach. However, because there was inadequate proof of superiority, these types of classifications had little effect on standard diagnostic techniques. It has been demonstrated in recent research that nuclear magnetic resonance (NMR) spectroscopy can be easily used in clinical laboratories to differentiate HDL into different size fractions [56–58]. This technique provides the large-to-small HDL ratio, which may be helpful in determining insulin resistance and other related disorders in addition to measuring the risk of CVD. HDL subfractions measured by NMR spectroscopy capture central features of circulating HDL that are not solely determined by apoA1 concentration or activity. Although apoA1 remains the dominant structural protein of HDL, particle-based metrics reflect additional compositional and functional attributes, including lipid content, accessory apolipoproteins and enzyme cargo. The observed associations between HDL particle metrics and cardiovascular outcomes may therefore likely arise from apoA1 independent mechanisms as well, including enhanced cholesterol acceptance by mature HDL particles and modulation of antioxidative and anti-inflammatory pathways. By prioritizing the investigation of HDL functionality and particle size, we can improve our understanding of HDL biology and open the door for more successful approaches for preventing and treating CVD [55, 59].

## Antioxidative and Anti-inflammatory Functions of HDL: Evidence Beyond Cholesterol Efflux

In addition to cholesterol efflux capacity, HDL exerts cardioprotective effects through antioxidative and anti-inflammatory mechanisms that contribute to vascular homeostasis and atheroprotection. These functions are mediated by HDL associated enzymes, apolipoproteins and lipid components and have been evaluated using a range of biochemical and cellular assays [60–62]. The antioxidative capacity of HDL is commonly assessed by its ability to inhibit low density lipoprotein (LDL) oxidation, often measured using cell free oxidation assay or endothelial cell models [63]. PON1, an HDL associated enzyme, plays a central role in this process by hydrolyzing oxidized lipids and preventing oxidative modification of LDL [63, 64]. Clinical studies have demonstrated that reduced HDL associated PON1 activity is associated with increased oxidative stress and higher cardiovascular risk, particularly in conditions such as diabetes mellitus, chronic kidney disease and established coronary artery disease [65–67].

Anti Inflammatory properties of HDL are evaluated by assessing its capacity to suppress endothelial adhesion molecule expression, reduce monocyte chemotaxis and modulate cytokine production in vascular and immune cells [68, 69]. Dysfunctional HDL isolated from patients with inflammatory states has been shown to lose these protective effects and, in some cases, to acquire pro-inflammatory properties. Observational studies have linked impaired anti-inflammatory HDL function with endothelial dysfunction and adverse cardiovascular outcomes, although such associations are less extensively characterized than those observed for CEC [70].

Compared to CEC, assays assessing antioxidative and anti-inflammatory HDL functions demonstrate greater methodological heterogeneity and limited standardization, which may partly explain variability in reported associations with cardiovascular outcomes [71]. Nevertheless, evidence suggests that these functions provide c complementary information regarding HDL quality and may be particularly relevant in disease characterized heightened oxidative stress and systemic inflammation [72].

Taken together, while CEC remains the most extensively validated functional metric of HDL, antioxidative and anti-inflammatory properties represent integral component of HDL biology. A comprehensive assessment of HDL functionality that integrates efflux capacity with these additional protective mechanisms may offer a more complete understandings of HDL’S role in cardiovascular disease [67, 69, 73].

## Comparative Assessment of HDL Functional Assays: Strengths, Limitations and Clinical Applicability

A few assays have been developed to evaluate HDL functionality; however, these methods differ substantially in their biological focus, technical complexity, reproducibility and clinical applicability. As a result, findings across studies are not always directly comparable and each assay must be interpreted within its methodological context [70, 71].

CEC assays are the most widely studied functional measure of HDL and are considered a robust surrogate of reverse cholesterol transport. Cell based models using macrophage lines such as J774 or THP-1 cells are HDL mediated cholesterol removal through ABCA1-1, ABCG1 and SR-BI dependent pathways [66, 71]. Despite their strong biological relevance, CEC assays exhibit considerable inter laboratory variability, influenced by differences in cell lines, cholesterol labeling techniques, stimulation protocols and use of apoB depleted serum versus isolated HDL. In addition, these assays are not easily scalable, limiting their routine clinical application.

Antioxidant and anti-inflammatory assays, including the assessment of HDL’s ability to inhibit LDL oxidation or suppress endothelial adhesion molecule expression. However, these assays lack methodological standardization and are highly sensitive to experimental conditions, oxidative substrates and inflammatory stimuli [65, 67]. Consequently, their reproducibility across laboratories is limited and their predictive value for cardiovascular outcomes remains less well established compared to CEC [72]. Enzyme activity-based assays, such as measurements of paraoxonase-1(PON-1) or LCAT activity, provide insight into specific components of HDL function. While these assays are relatively simple and reproducible, they reflect only isolated aspects of HDL biology and do not capture the integrated functional capacity of the HDL particle [65, 68, 73].

In contrast. HDL size and particle concentration, particularly nuclear magnetic resonance (NMR) spectroscopy and Lipoprint analysis, offer a high throughput and clinically feasible approach to characterizing HDL heterogeneity. These techniques demonstrate good analytical reproducibility and scalability, making them suitable for large cohort studies. However, they provide structural rather than functional information and do not directly measure biological activity such as cholesterol efflux or antioxidative capacity [74, 75].

Taken together, no single assay fully captures the multifaceted functionality of HDL. Cell based functional assays are best suited for mechanistic and translational research, whereas HDL particle profiling techniques are more appropriate for epidemiological studies and clinical risk stratification. The lack of assay standardization and the complementary nature of available methods highlight the need for integrated approaches that combine functional and structural assessment to better define HDL- related cardiovascular risk (Table 2).

**Table 2:** ** Overview of Assays Evaluating HDL Functionality Description:** This table summarises commonly used assays for assessing high density lipoprotein(HDL)function and characteristics, highlighting their biological relevance, strengths, limitations and typical applications in experimental and clinical research

Assay	Function	Strengths	Limitation	Best use
CEC (cell based)	Efflux of cholesterol	Strong biological relevance	Labor-intensive, variable	Mechanistic studies
Antioxidant Assays	LDL oxidation inhibition	Captures non-RCT effects	Poor standardization	Experimental research
PON1/LCAT activity	Enzyme-specific function	Reproducible	Partial HDL function only	Adjunct markers
NMR/Lipoprint	Particle size & number	High-throughput, scalable	Indirect function	Clinical & population studies

## Redefining Standards: Why HDL Particle Measurement Sets the bar Higher than HDL-C?

The assessment of HDL particles has become a crucial advance in predicting cardiovascular risk, surpassing the traditional reliance solely on HDL levels. Individuals can differ in the quantity and shapes of lipoprotein particles. Thus, even though their cholesterol levels may be similar, their risk of cardiovascular disease can also differ. For purpose of assessing the risk of CVD, nuclear magnetic resonance (NMR) spectroscopy could provide a more accurate measurement of particle size and number.

Lipoprint technology is a cutting-edge and crucial tool for profiling HDL in the field of lipid analysis. Lipoprint provides a complete understanding of lipoprotein composition and function by precisely separating and quantifying lipoprotein subclasses using state-of-the-art electrophoresis technology [76]. In contrast to conventional techniques that offer limited insights into HDL subfractions, the exceptional sensitivity and resolution of Lipoprint enable the discernment of discrete HDL particles, such as HDL2b, HDL2a, and HDL3, all of which possess distinct physiological functions. Clarifying the complex interactions that exist between lipoprotein subtypes and cardiovascular risk requires this level of detail, which provides researchers and practitioners with practical knowledge for individualized risk assessment [77–79].

The cardioprotective functions of HDL in CVD are not effectively represented by HDL cholesterol levels. Therefore, evaluating other parameters linked to HDL may have a better prognostic value than evaluating HDL. The size distributions of lipoprotein particles are becoming more important in predicting CVD risk, which is why there is growing interest in examining them. It has been found in recent research that a larger HDL particle size, rather than just the HDL level, is independently linked to better total cholesterol efflux capacity [80]. Although, the individuals with type 1 diabetes have larger HDL particle sizes, total and ABCA1-independent CEC remains elevated. In addition, a study found that patients with stable coronary artery disease (SCAD) and type 2 diabetes mellitus (T2DM), have elevated concentrations of the mixed HDL subfraction and are more likely to suffer from cardiac events [81–83].

The importance of measuring HDL particles in providing a more comprehensive awareness of HDL functionality and its role in cardiovascular health is highlighted by recent research.

Studies have underscored the limitations of HDL as a stand-alone marker of cardiovascular risk, as it fails to capture the diverse functionalities of HDL particles. HDL particle measurement provides insight into the heterogeneity of HDL subtypes, which is influenced by variations in size, density, and composition, which affect their biological activities [84–86]. For instance, a larger HDL particle size has been associated with improved CEC, a main mechanism in the RCT pathway that plays a pivotal role in mitigating atherosclerosis and cardiovascular events [8].

Moreover, the development of analytical techniques, such as NMR spectroscopy and ion mobility, has made it possible to accurately quantify and characterize HDL particles. The evaluation of HDL subfraction distribution can be done with these techniques, considering particle size and composition, as well as HDL levels. Lipoprint System (Quantimetrix) is a high-resolution electrophoresis system used to quantify lipoprotein subfractions by polyacrylamide gel electrophoresis (PAGE), enabling separation and quantification of HDL, LDL, and VLDL subclasses.The Lipoprint system is a key tool in the development of customized approaches for the management and prevention of CVD because of its good precision in analysing HDL heterogeneity [86–88]. Lipoprint is at the forefront of this evolving understanding of lipid metabolism, driving innovation and accelerating advancements in the pursuit of optimal cardiovascular health [69]. Such granularity in analysis enables clinicians to identify individuals with dysfunctional HDL profiles, even in the presence of seemingly normal HDL levels.

Additionally, it has been shown in recent research that HDL particle measurement is a better prognostic indicator of cardiovascular outcomes than HDL levels. Reduced cardiovascular risk has been linked to elevated concentrations of certain HDL subfractions, such as the large HDL subfraction, in numerous populations, including those with T2DM and CVD [89, 90]. In conclusion, the measurement of HDL particles offers a more nuanced approach to cardiovascular risk assessment than does the estimation of only HDL levels. By delving into the intricate details of HDL subpopulations, can better identify individuals at risk and tailor interventions aimed at improving HDL functionality, ultimately leading to more effective prevention and management of CVD [13, 75].

Evidence linking HDL particle size and number to cardiovascular outcomes is derived largely from epidemiological studies, including population based cohorts and secondary analyses of clinical trials. These studies consistently demonstrate an inverse association between HDL particle concentration or mean particle size and cardiovascular risk. However, mechanistic evidence explaining how specific HDL size subclasses directly modulate atherogenesis remains limited. Experimental studies suggest that larger HDL particles may exhibit enhanced cholesterol efflux and antioxidative capacity, but these observations have not been conclusively shown to mediate cardiovascular protection in vivo [91].

The average size and quantity of HDL particles in circulation are two new indicators of CVD. A few methods, such as ion mobility assays, gradient gel electrophoresis, and NMR spectroscopy, are used to measure the concentration of HDL particles. The JUPITER trial and the MESA cohort research suggests that CVD risk is lower when there is a higher concentration of HDL particles [21, 92, 94]. HDL diversity can be comprehensively measured by determining the mean size of HDL particles, which can be achieved through techniques such as NMR or ion mobility assessments. A larger mean HDL size is associated with a lower risk of CVD. Further evidence suggests that the size of HDL particles affects paraoxonase activity and other HDL functions, such as CEC.

Taken together, mechanistic studies provide very strong biological plausibility for HDL functionality, particularly cholesterol efflux, as a protective process in atherosclerosis. However, much of the evidence linking HDL particle size, HDL functionality and its cardiovascular outcomes in humans remains associative rather than causal. This distinction is critical when interpreting clinical data and underscores the need for interventional studies and standardized functional assays to clarify the causal role of HDL-related metrics in cardiovascular risk reduction.

### Consistency of NMR- and Lipoprint Based HDL Subclass Findings Across Clinical Settings

Evidence derived from diverse clinical settings suggests that HDL subclass measurements obtained through NMR spectroscopy and Lipoprint analysis demonstrate both consistent patterns and context-specific variability in their association with cardiovascular outcomes. Large population-based cohort studies, including the MESA and JUPITER trial sub- analyses have consistently reported that higher HDL particle concentration as measured by NMR [90–92], is inversely associated with incident cardiovascular event, independent of HDL cholesterol levels. These associations have been observed across sex and ethnic subgroups, supporting the robustness of NMR-derived HDL particle metrics in primary intervention settings.

In contrast, studies conducted in individuals with established cardiometabolic disease revel more heterogenous findings. In patient with T2DM and stale coronary artery disease, altered HDL subclass distributions characterized by a relative reduction in large HDL particles and increase in smaller or intermediate subclasses have been associated with adverse cardiovascular outcomes. However, the magnitude and direction of these associations vary across studies, likely reflecting disease related remodeling of HDL particles, differences in glycemic control, inflammatory burden and lipid lowering therapy use.

Lipoprint based HDL subclass analyses have similarly demonstrated that reduced large HDL subfractions and a predominance of smaller HDL particles are associated with increased cardiovascular risk in patients with coronary disease and metabolic disorders. Nevertheless, Lipoprint findings appear more sensitive to clinical context, with stronger associations reported in secondary prevention cohorts compared to general population studies. Differences in analytical resolution, subclass definitions and study populations may contribute to variability between Lipoprint and NMR-derived results.

Collectively, these findings indicate that while HDL particle measurements obtained via NMR and Lipoprint exhibit consistent inverse associations with cardiovascular risk in population-based cohorts, their predictive performance is more variable in disease-specific clinical settings. This underscores the importance of interpreting HDL subclass data within the appropriate clinical context and highlights the need for harmonization of subclass definitions and outcome measures across platforms.

## Intermittent Fasting: A strategy of Debate to Improve HDL Function

A promising approach to improving HDL function and overall lipid metabolism is intermittent fasting(IF). It may influence HDL functionality through multiple interconnected metabolic pathways, including alterations in lipid flux, inflammatory signalling, oxidative stress and apolipoprotein metabolism. Rather than acting solely through changes in HDL circulating cholesterol, IF-induced metabolic adaptations may affect HDL particle remodelling and enzyme activity. IF has gained attention for its beneficial effects on weight management, insulin sensitivity, and dyslipidaemia. IF is a type of dietary pattern that involves alternate periods of eating and fasting, with fasting intervals typically lasting from 12 h to 3–4 days [93, 94]. This approach has garnered attention for its potential benefits on lipid metabolism and cardiovascular health. A quasi-randomized clinical trial by Ahmed et al. investigated the impact of a 12-hour daytime fasting regimen, conducted three times per week over six weeks, on lipid profiles in South Asian adults with sub-optimal HDL levels. The study showed a significant decrease in body weight, BMI, waist circumference, total cholesterol, and LDL cholesterol. Notably, there was a significant increase in HDL cholesterol levels, suggesting that IF may enhance cardiovascular health by improving lipid profiles [95].The observed increase in HDL cholesterol levels following IF in this study also reflects enhanced HDL particle remodelling and reduced TG enrichment of HDL, processes that are known to influence HDL stability and function. Weight loss and improved insulin sensitivity associated with IF could reduce CETP-Medicated lipid exchange and hepatic lipase activity, potentially favouring the formation of larger HDL. However, as HDL-C levels alone do not necessarily correlate with efflux, these findings should not be interpreted as definitive evidence of improved HDL function [93, 95]. A network meta-analysis was conducted by Semnani-Azad et al. to compare different IF strategies and continuous energy restriction on cardiometabolic outcomes. The findings indicated that alternate-day fasting (ADF) and continuous energy restriction both resulted in weight reduction. Furthermore, ADF showed advantages in decreasing LDL cholesterol in comparison to libitum diets and continuous energy restriction. However, the study did not find significant effects of ADF on HDL cholesterol levels [96]. The absence of consistent effects of alternate day fasting on HDL cholesterol levels observed in this study does not preclude potential effects on HDL functionality. IF related improvements in insulin sensitivity and systemic inflammation may preferentially influence HDL quality, without necessarily altering HDL-C concentrations.

Pammer et al. conducted a recent randomized controlled trial in individuals with type 2 diabetes mellitus (T2DM) and obesity. Over a 12-week period, the study evaluated the effects of IF combined with dietary recommendations. Results showed significantly elevated serum apolipoprotein M (apoM) levels, with no substantial changes in cholesterol efflux capacity, PON1 activity, or LCAT activity. Although, IF may influence apoM levels, its overall impact on other HDL functional metrics requires further investigation [97, 98]. Notably, the lack of significant changes CEC, PON1 activity and LCAT activity highlights that IF induced alterations in HDL composition may selectively affect specific functional pathways rather than globally improving HDL function. More long-term studies and randomized controlled trials are needed to confirm the reports of positive effects of IF on HDL-C levels, and understand the mechanisms involved.

## Conclusions and Future Directions

Emerging evidence increasingly supports the concept that HDL functionality rather than HDL cholesterol concentration alone and provides a more informative framework for cardiovascular risk assessment. Cholesterol efflux capacity, particularly through ABCA-1 mediated pathways, represents a key mechanistic component of reverse cholesterol transport and has demonstrated consistent inverse associations with cardiovascular outcomes. However, HDL mediated atheroprotection is multifactorial, encompassing antioxidative, anti-inflammatory and endothelial protective functions. Advances in HDL particle characterization using techniques such as nuclear magnetic resonance spectroscopy and Lipoprint analysis have further highlighted the structural heterogeneity of HDL and its relevance to cardiovascular risk. While HDL particle number and size show robust associative relationships with outcomes in population based studies, their predictive value appears more variable in cardiometabolic disease states, underscoring the importance of contextual interpretation and methodological harmonization across platforms. Importantly, both apoA-1-dependent and apo A-1 independent pathways contribute to HDL functionality and clinical associations. Metrics such as cholesterol efflux capacity and HDL particle profiling should therefore be viewed as integrative measures of the overall HDL pool rather than surrogates for isolated molecular mechanisms. Lifestyle mechanisms, including intermittent fasting, may modulate HDL functionality through indirect effects on metabolic regulation, inflammation, and HDL composition rather than uniform enhancement of efflux pathways. These observations reinforce the need to assess functional aspects of HDL endpoints when evaluating the cardiovascular impact of dietary and metabolic interventions.

## Key References

- Cordero A, Muñoz-García N, Padró T, Vilahur G, Bertomeu-González V, Escribano D, et al. HDL function and size in patients with on-target LDL plasma levels and a first-onset ACS. *Int J Mol Sci.* 2023;24:5391. 10.3390/ijms24065391.
  - ◦This study highlights that HDL functionality and particle size remain important determinants of cardiovascular risk even when LDL cholesterol targets are achieved. The findings emphasize the role of HDL quality, rather than only HDL quantity, in predicting outcomes following acute coronary syndrome.
- Zhang W, Jin J, Zhang H, Zhu Y, Dong Q, Sun J, et al. The value of HDL subfractions in predicting cardiovascular outcomes in untreated diabetic patients with stable coronary artery disease: an age- and gender-matched case-control study. *Front Endocrinol (Lausanne).* 2022;13:1041555. 10.3389/fendo.2022.1041555.
  - ◦This study demonstrates that HDL subfractions provide additional prognostic value for cardiovascular outcomes in diabetic patients with coronary artery disease. The work underscores the clinical relevance of HDL particle heterogeneity and supports the concept that HDL functionality and composition are important biomarkers in cardiometabolic risk assessment.
- Mehta A, Shapiro MD. Apolipoproteins in vascular biology and atherosclerotic disease. *Nat Rev Cardiol.*2022;19:168–179. 10.1038/s41569-021-00613-5.
  - ◦This comprehensive review provides critical insights into the role of apolipoproteins in regulating lipoprotein metabolism and vascular biology. It highlights how different apolipoproteins influence atherosclerosis development and discusses emerging therapeutic strategies targeting apolipoprotein pathways to reduce cardiovascular risk.

## Data Availability

No datasets were generated or analysed during the current study.

## Declarations

### Transparency statement

The corresponding author affirms that this manuscript is a honest, accurate, and transparent account of the study being reported; that no important aspects of the study have been omitted; and that any discrepancies from the study as planned (and, if relevant, registered) have been explained. All authors have read and approved the final version of the manuscript and takes complete responsibility for the integrity of the data presented. 

### **Review methodology**

This article was conducted as a narrative review aimed at foundational concepts and recent advances related to HDL function, particle size and its cardiometabolic implications. A narrative review approach was selected to allow an integrative and interpretive discussion of mechanistic, clinical and translational evidence in a conceptually heterogeneous and rapidly evolving field. A comprehensive literature search was performed using PubMed, Scopus, Google Scholar databases. Inclusion criteria comprised peer reviewed original research articles, revies and clinical studies that addressed HDL biology, functionality and particle heterogeneity. Both experimental and clinical studies were considered to provide a comprehensive overview. Exclusion criteria included conference abstracts, non-peer reviewed articles, case reports and articles not directly relevant to the objectives of this review. The selected literature was synthesised thematically and emphasis was placed on integrating mechanistic insights with clinical findings to highlight emerging perspectives on HDL functionality beyond traditional HDL cholesterol measurements. 
